# DUAL PRIORITIES: DIGITAL TWINS AND HEALTH CARE DESIGN THAT SUPPORTS OLDER ADULTS IN THE CONTEXT OF STAFFING SHORTAGES

**DOI:** 10.1093/geroni/igae098.2022

**Published:** 2024-12-31

**Authors:** Felix Kabo, Jake Morrison, Chad Ritsema

**Affiliations:** CannonDesign, Ann Arbor, Michigan, United States; CannonDesign, Pittsburgh, Pennsylvania, United States; CannonDesign, Chicago, Illinois, United States

## Abstract

Aging populations are associated with a growing need and demand for specialized care, including healthcare environments that accommodate unique needs such as mobility assistance, chronic disease management, post-acute care, and mental health support. Currently, there are more than 46 million Americans aged 65 or over, and this number is projected to double by 2060. This demographic aging calls for an increased focus on design solutions like creating age-friendly spaces and ensuring accessibility. Designing healthcare environments that better support older adults is complicated by the staffing shortages in the American healthcare system. For example, there are significant projected shortages of both essential support staff (over 3 million in the next five years) and physicians (roughly 140,000 by 2033). These staffing shortfalls pose critical challenges to patient care, workplace culture and cohesion, and safety. The design of healthcare environments that better support older adults must mitigate the negative impacts of inadequate staffing levels and facilitate more efficient workflows e.g., enhancing interdisciplinary staff collaboration, promoting novel training opportunities, etc. This presentation examines the use of digital twins, effectively indistinguishable virtual representations of real-world physical objects (e.g., the human body, buildings, etc.), as part of a holistic design process that considers older adult patients, staffing and care delivery, and facility optimization. Digital twins have high fidelity and are linked to real-time data. They hold significant potential for designing age-friendly healthcare environments, optimizing staff and resource allocation, asset tracking, accelerated risk assessment, real-time decision support, and novel training simulations, thus improving care for older adults.

